# Suppressive effects of plumbagin on the growth of human bladder cancer cells via PI3K/AKT/mTOR signaling pathways and EMT

**DOI:** 10.1186/s12935-020-01607-y

**Published:** 2020-10-27

**Authors:** Renjie Zhang, Zijian Wang, Wenjie You, Fengfang Zhou, Zicheng Guo, Kaiyu Qian, Yu Xiao, Xinghuan Wang

**Affiliations:** 1 Department of Urology, Zhongnan Hospital of Wuhan University, Wuhan, 430071 People’s Republic of China; 2 Department of Biological Repositories, Zhongnan Hospital of Wuhan University, Wuhan, 430071 People’s Republic of China; 3 Cancer Precision Diagnosis and Treatment and Translational Medicine, Hubei Engineering Research Center, Zhongnan Hospital of Wuhan University, Wuhan, 430071 People’s Republic of China; 4 Research Center of Wuhan for Infectious Diseases and Cancer, Chinese Academy of Medical Sciences, Wuhan, 430071 People’s Republic of China; 5 Department of Biomedical Engineering, School of Basic Medical Sciences, Wuhan University, Wuhan, 430071 People’s Republic of China; 6 Department of Urology, The Central Hospital of Enshi Tujia and Miao Autonomous Prefecture, Enshi, 445000 People’s Republic of China

**Keywords:** Plumbagin, Bladder cancer, PI3K/AKT/mTOR, Cell cycle, Apoptosis

## Abstract

**Background:**

Novel chemotherapeutic drugs with good anti-tumor activity are of pressing need for bladder cancer treatment. In this study, plumbagin (PL), a natural plant-derived drug extracted from Chinese herbals, was identified as a promising candidate for human bladder cancer (BCa) chemotherapy.

**Methods:**

The anti-tumor activity of PL was evaluated using a series of in vitro experiments, such as MTT, transwell assay, flow cytometry, quantitative real-time PCR (qRT-PCR) and western blotting. We established xenograft tumors in nude mice by subcutaneous injection with the human bladder cancer T24 cells.

**Results:**

The results showed that PL could inhibit the proliferation, migration and survival of BCa cells (T24 and UMUC3 cells) in a time- and dose-dependent way. We found PL promotes the cell cycle arrest and apoptosis by inhibiting PI3K/AKT/mTOR signaling pathway, which inhibits cell proliferation. In vivo, anti-tumor activity of PL was further investigated using a BCa cell xenograft mice model. To simulate clinical chemotherapy, the PL were intravenously injected with a dose of 10 mg/kg for 10 times. Compared with the blank control, the tumor weight in PL treated group decreased significantly from 0.57 ± 0.04 g to 0.21 ± 0.06 g (*P* < 0.001).

**Conclusions:**

In our study. We found PL inhibits the proliferation of T24 and UMUC3 cells in vivo and in vitro, which may play a role through several downstream effectors of PI3K/AKT/mTOR signaling pathway to promote the cell cycle arrest and apoptosis. Meanwhile, we consider that PL may inhibit the migration of bladder cancer cells via EMT suppression and induce ROS generation to make cell apoptosis. This work screened out a novel chemotherapeutic drug (plumbagin) with relatively good anti-tumor activity, which possessed great potential in BCa chemotherapy.

## Introduction

Bladder cancer (BCa) is the most common urological carcinoma with more than 4.3 × 10^5^ new cases per year [1, 2]. BCa is complex in histopathological characteristics and is usually divided into two independent groups including muscle invasive and non-muscle invasive BCa [3]. The natural storage of urine in the bladder makes intravesical administration the most effective route of medication and treatment besides surgical resection, which has led to researchers have never stopped exploring new drugs for bladder cancer. A distinctive biological reagent named as Bacille Calmette-Guérin (BCG) vaccine also received great success in bladder cancer treatment. European Association of Urology (EAU) has recommended BCG bladder irrigation as one of the preferred adjuvant treatments for non-muscle invasive bladder cancer in 2018 [4]. Despite extensive research achievements have been successfully applied, the survival of BCa patients is still not fully satisfactory. The major factor is toxic side effects on off-target organs with the treatment of disease. It has been reported that the 5-year mortality rate of the muscle invasive BCa patients with lymph node (LN) metastasis is more than 77% [5]. Thus, finding a new antineoplastic drug with higher bioavailability is currently urgent.

Plant-derived drugs (PDDs) have been used to anti-cancer applications for thousands of years [6–8]. Our group has screened out a series of BCa chemotherapeutic PDDs, and their anti-tumor mechanisms were also investigated. For instance, capsaicin could suppress the tumorigenesis of BCa xenograft in vivo via FOXO3a mediated pathways [9]. In general, our previous reports revealed that PDDs extracted from Chinese herbal medicine are effective for BCa chemotherapy. PL (5-hydroxy-2-methyl-1,4-naphthoquinone, C_11_H_8_O_3_) is a kind of hydroxyl-naphthoquinone which was extracted from the *Plumbago* genus plants. It is reported a variety of remarkable pharmacological properties that include antioxidant [10], anti-inflammatory [11], antifungal [12], antibacterial [13], antidiabetic [14] and neuroprotective properties [15]. Importantly, it exhibits its therapeutic potential in various types of malignant tumors in recent years [16–18]. Recent studies conducted PL could enhance its therapeutic efficacy through multiple drug delivery systems in cancer therapy [19]. However, the anti-tumor effect and mechanism of PL on BCa have rarely been investigated.

Phosphatidylinositol 3-kinase (PI3K) plays many important biological functions in cell proliferation, cell growth, differentiation and apoptosis. Many studies have illustrated that the PI3K signaling pathway could be abnormally activated in multiple types of cancer [20–22]. Various growth factors and signal transduction complexes activate and phosphorylate the receptor tyrosinase to recruit the p85 subunit of PI3K, phosphorylation of phosphatidylinositol is catalyzed and transformed phosphatidyl inositol triphosphate (PIP3). PIP3 activates AKT (Protein kinase B, PKB) and PDK1 (phosphoinositide dependent kinase- 1) by binding to their PH domains. PDK1 promotes phosphorylation of PKB/AKT at Ser308. The activated AKT phosphorylates various protein kinases and mediates transcription factors, the mammalian target of rapamycin (mTOR) [23], an important downstream in PI3K and AKT signal pathway, can regulate tumor cell proliferation and metastasis.

In this study, PL is the first identified as a potential chemotherapeutic PDD for BCa treatment. The anti-tumor activity and mechanism of PL are characterized by a series of in vitro experiments, such as MTT assay, transwell assay, flow cytometric analysis, qRT-PCR, as well as western blotting. We found PL induces the cell cycle arrest and apoptosis through regulating several downstream effectors of the PI3K/AKT/mTOR signaling pathway to further inhibit the proliferation of tumor cells. Meanwhile, we considered that PL may inhibit the migration of bladder cancer cells via EMT suppression and induce cancer cell apoptosis via ROS generation [24]. A xenograft animal model is further applied to evaluate the anti-tumor activity in vivo. Our results confirm that PL is able to inhibit the tumorigenesis of BCa. This work has successfully screened out a prospective PDD, and its application potential remains to be verified by subsequent clinical trials.

## Material and methods

### Reagents and chemicals

Commercial PL (C_11_H_18_O_3,_ MW:188.18, purity > 95%) and the fluorescent probe 2′,7′-dichlorofluorescin diacetate (DCFH-DA) were obtained from Sigma-Aldrich Biochemical Co., Ltd. (Bermuda, USA). Cell cycle staining kit was purchased from Multi Sciences Co., Ltd. (Zejiang, China), and Annexin V-FITC/PI apoptosis staining kit was purchased from BD Biosciecnes Co., Ltd. (San Jose, USA). Transwell chambers were purchased from Corning Co., Ltd. (New York, USA). Dimethyl sulfoxide, crystal violet, absolute ethanol, methanol and 4% paraformaldehyde solution were purchased from Sinopharm Co., Ltd. (Shanghai, China). Cell mediums including RPMI-1640 and DMEM (+ high glucose) were purchased form Biocreative Co., Ltd. (Wuhan, China). Fetal bovine serum, RNase-free water, penicillin, streptomycin, trypsin–EDTA solution were purchased from Thermofisher Scientific Co., Ltd. (Waltham, MA, USA). All other chemicals were of analytic purity and applied as received.

### BCa cell lines

293 T cell lines, HK-2 cell lines, human epithelial SV40 immortalized uroepithelium cell line (SV-HUC-1) and human BCa cell lines (T24 and UMUC3) were obtained from the Stem Cell Bank, Chinese Academy of Sciences in Shanghai, China. Two kinds of bladder cancer cell lines were used in this work. All cell lines were identified by STR profiling before use. SV-HUC-1 cells and T24 cells were cultured in complete medium composed of 90% RPMI-1640 medium, 10% fetal bovine serum (FBS) and 1% penicillin/streptomycin stocking solution. 293 T cells, HK-2 cells and UMUC3 cells were also cultured in DMEM complete medium. Additionally, a 37 ℃ incubator filled with 5% CO2 atmosphere was used for cell incubation.

### Cell viability assay

PL was firstly dissolved into dimethylsulfoxide (DMSO) with constant stirring to prepare a PL stocking solution with a concentration of 100 μg/mL, and then diluted with complete medium to obtain the PL working solution with a concentration of 0, 0.25, 0.5, 0.75, 1.0 and 1.5 μM, respectively. To screen out the appropriate drug concentration for in vitro tests, MTT assay was performed. Briefly, BCa cell lines were seeded into 96 well tissue culture plates with the cell concentration of 4.0 × 10^4^ /mL. After the cells attached to the plates for 12 h, the complete medium in each well was removed and replaced with fresh PL working solution. After further incubated for 24 and 48 h, 20 μL of MTT working solution was dropped into each well, and then incubated for another 4 h. After that, the solution in the 96 well tissue culture plate was removed, and 150 μL of DMSO was added with constant stirring. The optical density (OD) at 490 nm was measured by a microplate reader (SpectraMax^@^M2, MD, USA). The relative killing rate was calculated from at least three independent samples. To evaluate the anti-proliferation activity of PL against BCa cell lines, the T24 and UMUC3 cells were treated with PL working solution for sequential 4 days. At regular time intervals, the cells were treated with MTT reagent and the proliferation curves were determined.

### Flow cytometry analysis

The cell cycle staining analysis was performed to investigate the anti-proliferation activity of PL against BCa cell lines. BCa cells were firstly treated with PL working solution for 24 h, harvested into 1.5 mL centrifuge tubes for cell cycle staining. 1 mL of 1 × DNA staining buffer and 10 μL of permeabilization solution were added into each tube, and then re-suspended thoroughly. After further incubated at dark room for at least 30 min, the cell cycle distribution of treated cells was detected using a flow cytometry (CytoFLEX, Beckman, USA). The cell apoptosis staining was also carried out using an Annexin V-FITC apoptosis staining kit. The treated BCa cells were re-suspended into 1 mL of 1 × binding buffer, and then stained with 5 μL of Annexin V-FITC solution for 15 min and 5 μL of PI solution for another 10 min. The positive cell apoptosis rate was detected using a flow cytometry (CytoFLEX, Beckman, USA). The data was calculated from at least three independent samples. The ROS detection kit (Invitrogen) was applied to detect the level of intracellular ROS. The cells with treated PL were stained with 10 μM 2,7-dichlorofluorescin diacetate (DCFH-DA) for 30 min at 37 °C and washed twice with phosphate buffer saline (PBS) The fluorescence intensity of ROS level was recorded by flow cytometry.

### Clonogenic survival assay

BCa cells were seeded onto 6 well tissue culture plates, the cell number in each well was adjusted to 800. After these cells attached to the plates for 12 h, the complete medium in each well was removed and replaced with fresh PL working solution. The treated cells were cultured in an incubator for 10–15 days, until the cell clones became visible to naked eyes. The cell clones were fixed with 4% paraformaldehyde solution for 30 min, and then staining with 0.1% crystal violet solution for another 30 min. The residual dye was washed away using distill water, and the cell clones were captured using a using a digital camera (A7RIII, Sony, Japan). The clone number in each field was counted and at least three independent fields were used for statistical analysis.

### Cell migration assay

In this work, the migration ability of PL treated cells was evaluated using transwell assay and wound healing assay at the same time. For transwell assay, BCa cells were re-suspended with FBS-free PL working solution, and then seeded onto the upper layer of transwell chambers. The volume of medium was set to be 200 μL, and the cell number per chamber was set to be 4 × 10^5^. 700 μL of complete medium was added into the lower layer of transwell chamber. After incubated overnight, the treated cells were fixed with 4% PFA solution for 30 min, and then stained with 0.1% crystal violet solution for another 30 min. A cotton swab was applied to clean out the residual cells on the upper layer of transwell chamber. The photographs of migrated cells were captured using an inverted fluorescence microscope (IX73, OLYMPUS, Japan). the cell number per field was counted using the IPP-6.0 software, at least three independent samples were used for statistical analysis.

### Wound healing assay

BCa cells were seeded onto 6 well tissue culture plates with a relative high cell density. The cells were incubated overnight until the confluence reached over to 80%. A 200 μL tip was applied to scratch the tissue culture plates, and then a linear cell wound was fabricated. After rinsed with PBS solution for three times, these unattached cells were removed. 2.5 mL of PL working solution with 2% FBS was added into each well. At regular time intervals, the phototgraphs of wound site were captured using an inverted fluorescence microscope (IX73, OLYMPUS, Japan), and the wound healing rate was calculated from three independent samples.

### Quantitative real-time PCR

The BCa cells were treated with PL working solution for 24 h, and then the total RNA was harvested using a commercial HiPure RNA Kit (Magen, China). The experiment was performed in accordance with manufacturers’ protocols [25], and then the concentration of obtained RNA was detected using a nanodrop spectrophotometer (Thermo, USA). After that, 1 g of RNA was applied for the reverse transcription reaction, and the resultant cDNA were further applied for quantitative real-time PCR (qRT-PCR) analysis. In this work, iQTM SYBR®Green Supermix was obtained from Bio-Rad Co., Ltd. (Shanghai, China). The primer sequences for Murine Double Minute 2 (MDM2), growth arrest and DNA damage (GADD), p53, CDK2, CDK4, CDK6 and p21 were listed in Additional file 1: Table S1.

### Western blotting analysis

BCa cells were treated with PL for 24 h, and then used for western blotting test. Briefly, the cells were firstly immersed into 500 μL of RIPA buffer containing with 10 μL of phosphatase inhibitor and 10 μL of protease inhibitor. After lysed at 4 °C for 30 min, the mixed solution was centrifuged at 1.2 × 10^4^ g for 15 min. The supernatant was carefully harvested, and the concentration of protein was detected using an enhanced BCA assay kit (Abcam, China). The obtained protein samples were denatured at 95 ℃ for 10 min, and then separated by 10% sodium dodecyl sulfate polyacrylamide gel electrophoresis (SDS-PAGE) and electro-transferred onto polyvinylidene fluoride (PVDF), The PVDF samples were blocked by 5% fat-free milk solution for 2 h at room temperature and exposed to primary antibodies for 10 h at 4 °C, the PVDF were washed three times for approximately 10 min and incubated with the secondary antibodies for 2 h at room temperature, the PVDF were washed three times for approximately 10 min again. The protein bands were visualized using a enhanced chemiluminescence (ECL) kit. The ChemiDoc™ MP Imaging System (Bio-Rad, USA) was also used for data processing. The suppliers of primary antibodies and secondary antibodies applied in this study are listed in Additional file 1: Table S2.

### In vivo animal evaluations

This work was performed under supervision for the declaration of Helsinki and “Guiding Opinions on the Treatment of Animals” in China. The Ethics Committee at Zhongnan Hospital of Wuhan University has approved this experimental protocol. Specific pathogen free (SPF) BALB/C57 nude mice, 3–4 weeks old, were obtained from WTLH Co., Ltd. (Beijing, China). Before experiments, all mice were quarantined in animal experiment center for 7 days to relieve mental stress.

BCa T24 cells were digested and harvested with PBS, the cell density was then adjusted to 1.5 × 10^7^ cells/mL. The mice were carefully sterilized using 75% alcohol solution for three times, and then injected with 200 μL of cell suspension. After that, all animals were quarantined for another 10–15 days until the tumors become visible to naked eyes. PL was dissolved into ethanol with a concentration of 10 mg/mL, and then diluted using PBS to prepare PL working solution. Three animals were injected with PL working solution at a dose of 10 mg/Kg. They were treated every third day for 10 times. For the control group, three animals were treated with ethanol solution with equal dose. Tumors’ size was detected using Vernier caliper, and tumor volume (mm^3^) was calculated as A_L_ × A_W_^2^ × 0.5, where A_L_ and A_W_ respectively refer to the length and width of tumors. After the drug injection was finished, all tumor and organ samples including heart, liver, spleen, lung and kidney, were dissected, and then immersed into sufficient 4% paraformaldehyde solution for at least 72 h. Histological analysis including HE and Masson was performed according to the manufacturer’s protocols.

### Hematoxylin and eosin (H&E) and masson staining

Three pairs of tumor tissue paraffin sections and normal organic tissue (heart, liver, spleen, lung and kidney) were stained with Hematoxylin and Eosin (H&E). All slides were deparaffinized, rehydrated in xylene, 100%, 96%, 80%, 70% ethanol and H2O, and constantly stained with 10% hematoxylin (Sigma-Aldrich) for 7 min, then the cytoplasm was stained by 1% eosin with 0.2% glacial acetic acid. 70%, 80%, 96%, and 100% ethanol and xylene were used to dehydrate for all slides. Heart, liver, spleen, lung and kidney were stained with Masson staining by a Masson’s Trichrome Staining Kit (Solarbio Science & Technology, Beijing) to show the extent of inflammatory repair and fibrosis. Images were taken by an inverted phase contrast microscope (Leica, Cat. #DMI 1).

### Statistical analysis

The data was collected from no less than three independent experiments, and the statistical results were shown as mean ± standard deviation. One-way ANOVA and post hoc Tukey’s test was used for statistical analysis. *P* < 0.05 was set to be statistically different. Statistical significance between two different drug concentrations’ groups are as follows: * *P* < 0.05, ** *P* < 0.01, and *** *P* < 0.001.

## Results

### Screening PL concentration for in vitro evaluation

**Fig. 1:**
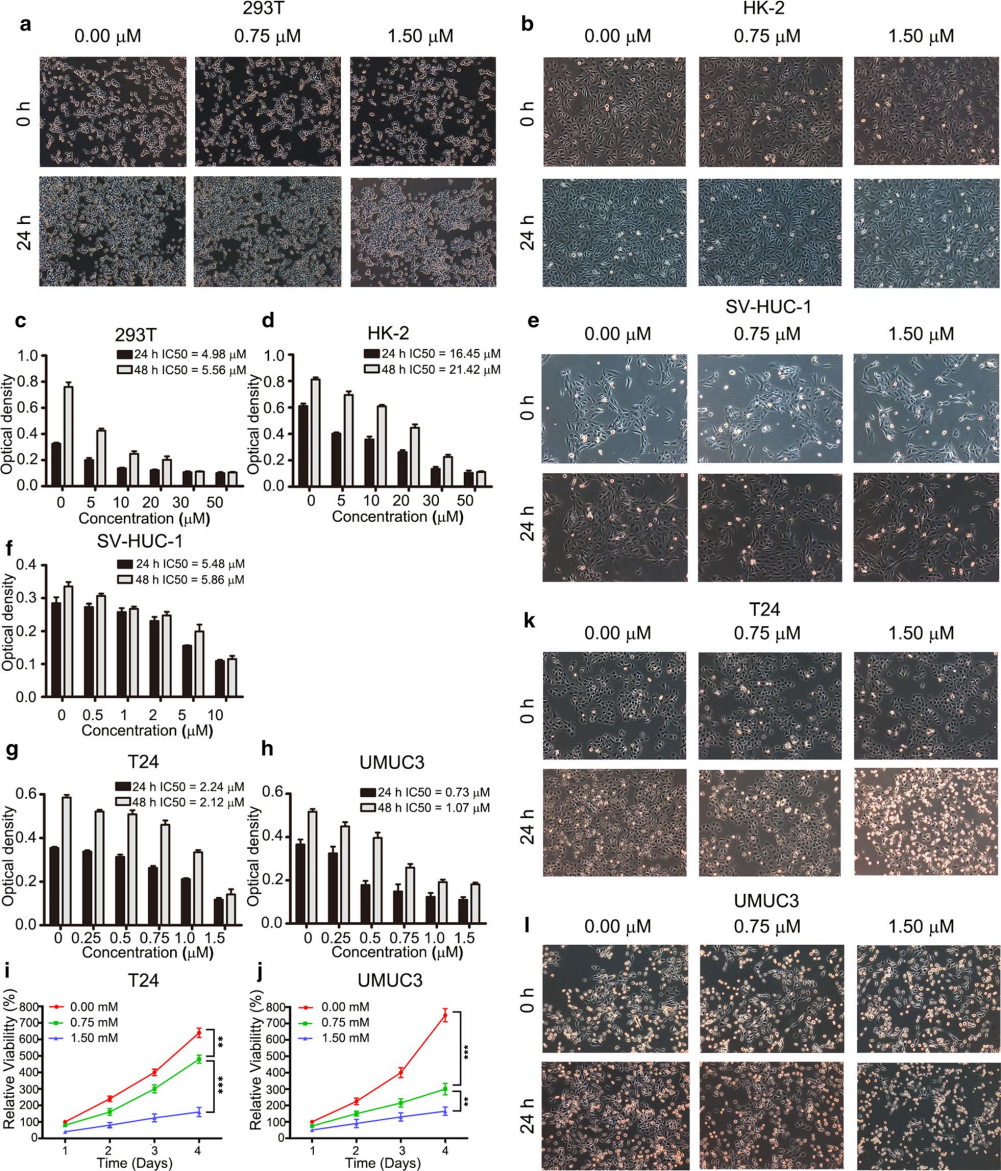
Plumbagin inhibited the proliferation of BCa cells rather than normal cells. The representative images of 293 T cells (**a**) and HK-2 cells (**b**) were treated with 0, 0.75 and 1.5 μM in 24 h, 293 T cells (**c**) and HK-2 cells (**d**) cells were cultured with the concentration gradient of plumbagin working solution (0, 5, 10, 20, 30, 50 μM) in 24 h and 48 h; the representative images of SV-HUC-1 cells were treated with the concentration (0, 0.75, 1.5 μM) (**e**) in 24 h and the concentration (0, 0.5, 1, 2, 5, 10 μM) in 24 h and 48 h (**f**); T24 cells (**g**) and UMUC3 cells (**h**) were treated with the concentration (0, 0.25, 0.5, 0.75, 1, 1.5 μM) in 24 h and 48 h, the proliferation curves of T24 cells (**i**) and UMUC3 cells (**j**), which were treated with plumbagin working solution (0, 0.75, 1.5 μM) for consecutive 4 days, the representative images of T24 cells (**k**) and UMUC3 cells (**l**) were treated with the concentration (0, 0.75, 1.5 μM) in 24 h. Magnification Power: 100×. **P < 0.01, ***P < 0.001

In this work, MTT assay was performed to screen out the appropriate PL concentration for the following experiments. Briefly, multiple normal cell lines (293 T, HK-2 and SV-HUC-1 cell lines) and BCa cells (T24 and UMUC3) were firstly treated by PL working solutions for 24 or 48 h. The concentrations of PL working solutions in 293 T cells and HK-2 cells were set to be 0, 5, 10, 20, 30 and 50 μM, the concentrations of PL working solutions in SV-HUC-1 cells were set to be 0, 0.5, 1, 2, 5 and 10 μM, the concentrations of PL working solutions in T24 and UMUC3 cell were set to be 0, 0.25, 0.5, 0.75, 1.0 and 1.5 μM. After that, the optical density of each sample was detected at a wavelength of 490 nm. As shown in Fig. 1, the optical density of all cells decreased gradually along with an increase of PL concentration. The value of inhibitory concentration 50 (IC50) was further calculated. In normal cells, for 293 T cells, the value of IC50 was 4.98 μM after 24 h treatment and 5.56 μM after 48 h treatment in Fig. 1c, for HK-2 cells, the value of IC50 was 16.45 μM after 24 h treatment and 21.42 μM after 48 h treatment in Fig. 1d, for SV-HUC-1 cells, the value of IC50 was 5.48 μM after 24 h treatment and 5.86 μM after 48 h treatment in Fig. 1f. In BCa, for T24 cells, the value of IC50 was 2.24 μM after 24 h treatment and 2.12 μM after 48 h treatment in Fig. 1g. For UMUC3, the results of MTT assay exhibited a similar tendency. The value of IC50 was 0.73 μM after 24 h treatment, and 1.07 μM after 48 h treatment in Fig. 1h. Based on the above results, we proposed to set three groups for the following experiments: high dose group (1.5 μM), low dose group (0.75 μM) and blank control group (0 μM). We further observed morphological changes in above five cells treated with three different concentrations by an inverted phase contrast microscope. Compared with the blank control group (0 μM), 293 T cells (Fig. 1a), HK-2 cells (Fig. 1b) and SV-HUC-1 cells (Fig. 1e) showed intact membrane and normal nuclear morphology after low (0.75 μM) and high (1.5 μM) concentration treatment. In contrast, for T24 cells (Fig. 1k) and UMUC3 cells (Fig. 1l), the plasma membrane ruptured, cytoplasmic content flowed out, and partial nuclear pyknosis, fragmentation and lysis were observed clearly.

### BCa cell proliferation was inhibited in a dose- and time-dependent way

The anti-proliferation activity of PL was further evaluated with extended treating time for consecutive 4 days. The proliferation curves of T24 and UMUC3 cells are respectively shown in Fig. 1i–j. The relative viability of treated cells decreased significantly as the concentration of PL increased. At the 4th day, the relative viability of T24 cell were 640 ± 28% for 0 μM group, 480 ± 24% for 0.75 μM group and 160 ± 26% for 1.5 μM group, respectively. Meanwhile, the relative viability of UMUC3 cell were 750 ± 30% for 0 μM group, 300 ± 25% for 0.75 μM group and 165 ± 25% for 1.5 μM group, respectively. A significant difference was observed between each group (*P* < 0.01). The anti-proliferation activity of PL was also enhanced as the treatment time was prolonged. At the same time, we also carried out a clonogenic survival assay to re-examine the effect of PL on the proliferation ability of cancer cells, the images are shown in Additional file 1: Fig. 1a and its’ quantitative results are shown in Additional file 1: Fig. 1b. For T24 cell, the number of cell clones was 253 ± 7 for the 0 μM group, and then significantly decreased to 106 ± 9 for the 0.75 μM group and 66 ± 4 for the 1.5 μM group, respectively (*P* < 0.001). For UMUC3 cell, the number of cell clones was 78 ± 3 for the 0 μM group, and then significantly decreased to 26 ± 2 for the 0.75 μM group and 8 ± 1 for the 1.5 μM group, respectively (*P* < 0.001). It was shown that PL reduced BCa cells survival effectively. In conclusion, PL inhibited the proliferation of BCa cells in a dose- and time-dependent manner.

### PL blocked cell migration via EMT

**Fig. 2:**
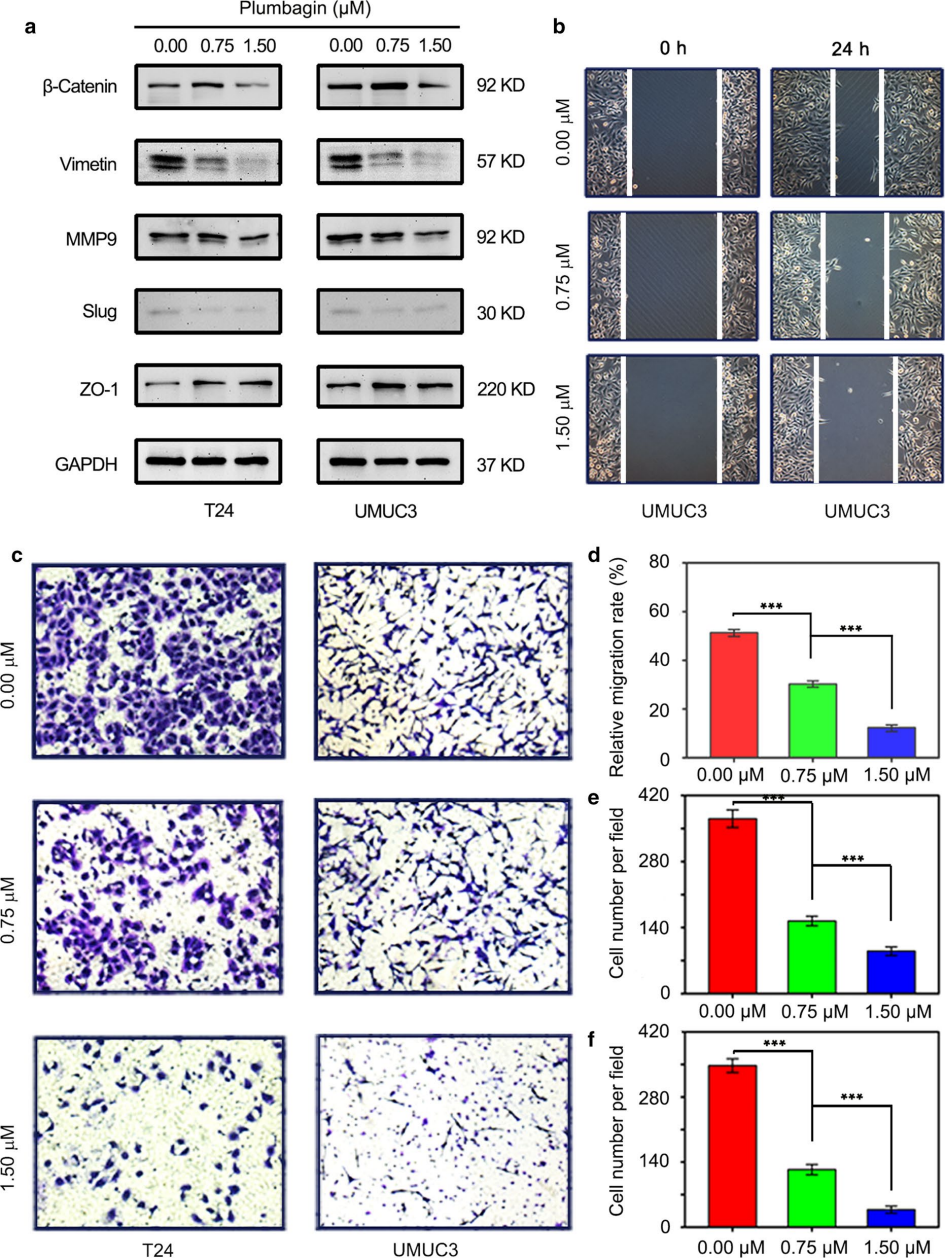
Plumbagin inhibited the migration of BCa cells by EMT suppression. **a** The expression levels of β-Catenin, Vimentin, MMP9, Slug and ZO-1 protein determined by western blotting assay when T24 and UMUC3 cells were treated with plumbagin at 0, 0.75, 1.5 μM. GAPDH was used as the internal control; **b** The anti-migration activity of plumbagin for UMUC3 cells was further evaluated by using wound healing assay; **c** Representative images of migrated BCa cells (T24 and UMUC3) after plumbagin treatment; **d** Quantitative analysis of wound healing assay for UMUC3 cells; Quantitative analysis of T24 **e** and UMUC3 (**f**) after plumbagin treatment for transwell assay. ***P < 0.001

The malignant proliferation and terminal migration of cancer cells are important biological behaviors of malignant tumors. We continued to explore the effect of PL on bladder cancer cell migration, the cell migration capability after PL treatment was investigated by transwell assay and wound healing assay at the same time. The representative images for transwell assay are shown in Fig. 2c. The migrated cells for both T24 and UMUC3 cells declined obviously after PL treatment. The quantitative results of transwell assay are shown in Fig. 2e–f. For T24 cell, the number of migrated cells in each field decreased from 366 ± 17% (0 μM group) to 147 ± 5 (0.75 μM group) and 92 ± 4 (1.5 μM group); for UMUC3 cell, the number of migrated cells in each field decreased from 351 ± 13 (0 μM group) to 123 ± 6 (0.75 μM group) and 38 ± 3 (1.5 μM group). A significant difference was observed between each group (*P* < 0.001). In would healing assay, the representative images for T24 and UMUC3 cells are respectively shown in Additional file 1: Figs. 1a and 2b, the corresponding statistical results of wound healing rate are shown in Additional file 1: Figs. 1d and 2d. This result was highly inconsistent with that of transwell assay. The EMT is an important pathway that affects the migration of cancer cells, the expressions of β-Catenin, Vimentin, MMP9, Slug and ZO-1 in T24 and UMUC3 cells were examined by Western blotting in Fig. 2a, after treatment with 0, 0.75 and 1.5 μM PL for 24 h, β-Catenin, Vimentin, MMP9, Slug had decreased on dependent-concentration, in contrast, ZO-1 had increased. In conclusion, the migration ability of BCa cells was significantly blocked by PL treatment by EMT suppression.

### PL induced G1 cell cycle arrest

**Fig. 3:**
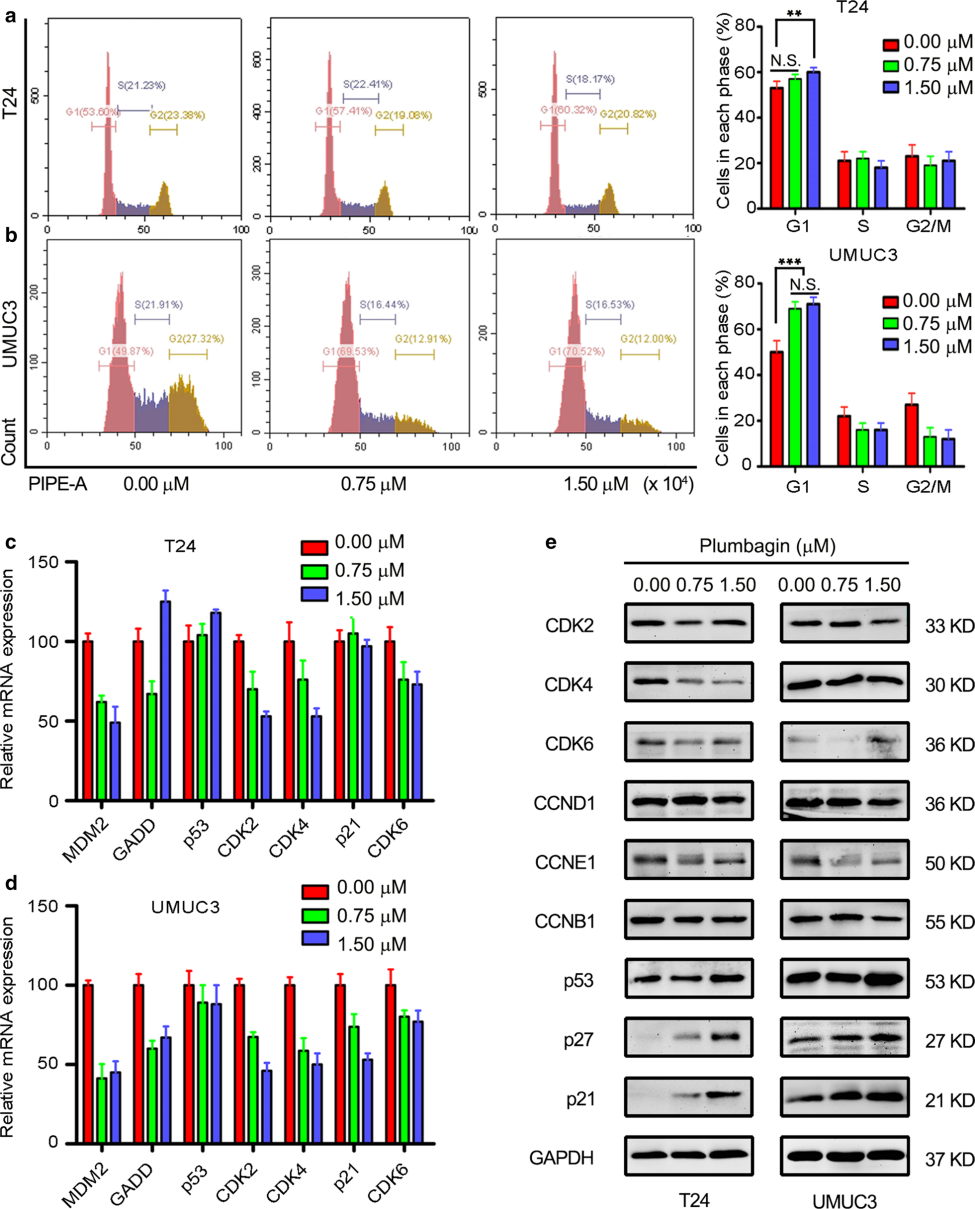
Cell cycle of BCa cells was arrested in G0/G1 phase by plumbagin treatment. **a**,** b** Representative FACS images of cell cycle with plumbagin treatment in T24 and UMUC3 cells, respectively, and quantitative analysis of cell cycle proportions showed a remarkable increase in G0/G1 phase cells; **c**,** d** The relative mRNA expression of MDM2, GADD, p53, CDK2, CDK4, p21 and CDK6 could be modulated when T24 and UMUC3 cells were treated with plumbagin at 0, 0.75, 1.5 μM; **e** The expression levels of CDK2, CDK4, CDK6, CCND1, CCNE1, CCNB1, p53, p27, p21 were determined by western blotting assay. GAPDH was as the internal control. **P < 0.01, ***P < 0.001

We preliminarily explored the mechanism of cell migration with PL treatment, next we mainly studied the inhibitory effect of PL on the proliferative activity of cancer cells. Cell cycle arrest, cell apoptosis, autophagy and other biological events are known to affect the proliferation of cancer cells, flow cytometry was applied to detect cell cycle. Representative FACS images of cell cycle assay are shown in Fig. 3a–b, the percentage of G0/G1 phase in both two kinds of BCa cells was obviously up-regulated after 24 h with PL treatment, the quantitative results of cell cycle proportions are shown in Fig. 2a–b. For T24 cell, the percentage of G0/G1 phase cells increased from 53.3 ± 1.6% (0 μM group) to 57.4 ± 1.3% (0.75 μM group) and 60.3 ± 0.8% (1.5 μM group), respectively; For UMUC3 cell, the percentage of G0/G1 phase cells increased from 49.8 ± 2.1% (0 μM group) to 69.5 ± 1.5% (0.75 μM group) and 70.5 ± 1.2% (1.5 μM group), respectively. A significant difference was observed between each group (*P* < 0.01). This phenomenon indicated that PL arrested cell cycle at G0/G1 phase. To uncover the molecular mechanism of PL’s this activity, qRT-PCR and western blotting were carried out. The expression of genes, such as GADD, MDM2, CDK2, CDK4, p53 and p21 was detected by qRT-PCR. As shown in Fig. 3c–d, MDM2, CDK2, CDK4 exhibited an obviously down-regulation tendency. The results of western blotting also indicated an obvious down-regulation of Cyclin (CCND1, CCNE1) and Cyclin-dependent kinase (CDK2, CDK4 and CDK6) proteins in a concentration-dependent in both T24 and UMUC3 cells (Fig. 3e) while the cyclin kinase inhibitor (p21, p27) was increased. But there was no significant change for CCNB1 in a concentration-dependent in both cells. This confirmed that the cell cycle arrest effect of PL was achieved through G0/G1 phase instead of G2 phase. This phenomenon has further confirmed that cell cycle was arrested by PL treatment, and then contributed to the anti-proliferation activity.

### PL promoted cell apoptosis by modulating key apoptotic regulators

**Fig. 4:**
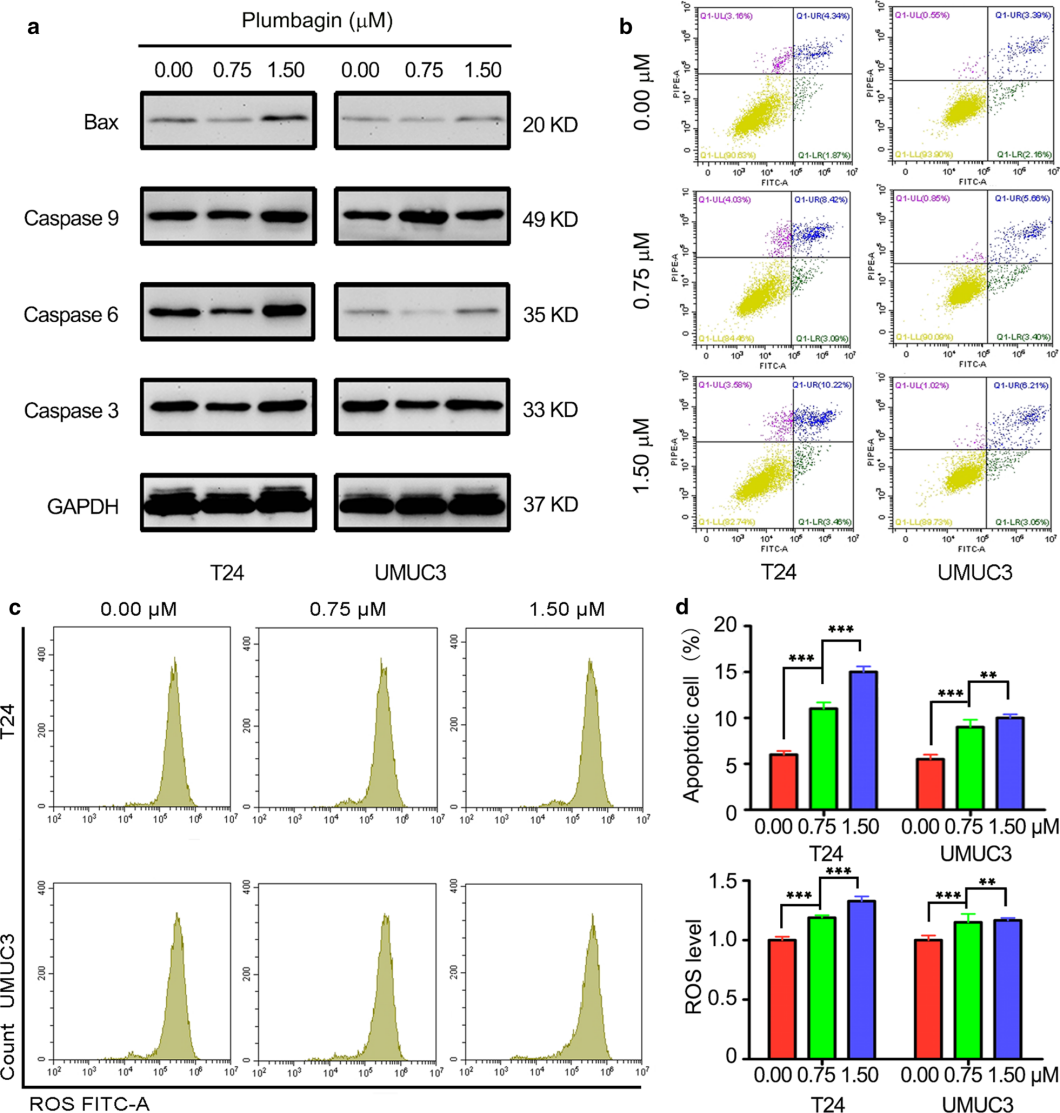
Plumbagin promoted BCa cells apoptosis and ROS generation. **a** The protein expression of BAX, Caspase 3, Caspase 6 and Caspase 9 was shown by western blotting; **b** representative FACS images of cell apoptosis in plumbagin treated T24 and UMUC3 cells; **c** representative FACS images of ROS in plumbagin treated T24 and UMUC3 cells; **d** quantitative results of (**b**); **e** quantitative results of intracellular ROS. **P < 0.01, ***P < 0.001

The next step is to detect cell apoptosis for exploring its intrinsic mechanism of inhibiting proliferation. The influence of PL treatment on BCa cell apoptosis was investigated using an apoptosis staining method by flow cytometry (FCM). The representative images of cell apoptosis are shown in Fig. 4b and the quantitative results of the apoptosis rate are shown in Fig. 4d. After treated with PL for 24 h, the percentage of apoptosis cell in both two kinds of BCa cells were significantly increased (*P* < 0.01). For T24 cells, the percentage of apoptosis cell increased from 6.2 ± 0.7% (0 μM group) to 11.3 ± 1.2% (0.75 μM group) and 14.4 ± 1.0% (1.5 μM group). For UMUC3 cells, the percentage of apoptosis cell increased from 5.5 ± 0.9% (0 μM group) to 9.1 ± 1.4% (0.75 μM group) and 9.2 ± 0.7% (1.5 μM group). Next, the expression of the protein, such as BAX, Caspase 3, Caspase 6 and Caspase 9 was detected by western blotting in Fig. 4a, the results of western blotting also indicated BAX, Caspase 3, Caspase 6 and Caspase 9 had increased in different degrees with different concentrations for 24 h. This suggests that PL can promote the apoptosis of tumor cells in a concentration-dependent way. In addition, we also detected intracellular ROS in drug-treated cells by FCM (Fig. 4c) and found that PL could induce cells to produce more ROS with the increase of concentration. In Fig. 4e, for T24 cells, compared with blank control group cells, the folds of ROS level had increased to 1.28-fold (0.75 μM group) and 1.42-fold (1.5 μM group). For UMUC3 cells, compared with blank control group cells, the folds of the ROS level had increased to 1.25-fold (0.75 μM group) and 1.27-fold (1.5 μM group). ROS generation may also be one of the pathways for PL-induced apoptosis. This is consistent with the research of Xue et al. [26].

### PLB suppresses the growth of the human bladder cancer cells via inhibiting PI3K/AKT/mTOR signal pathway

**Fig. 5:**
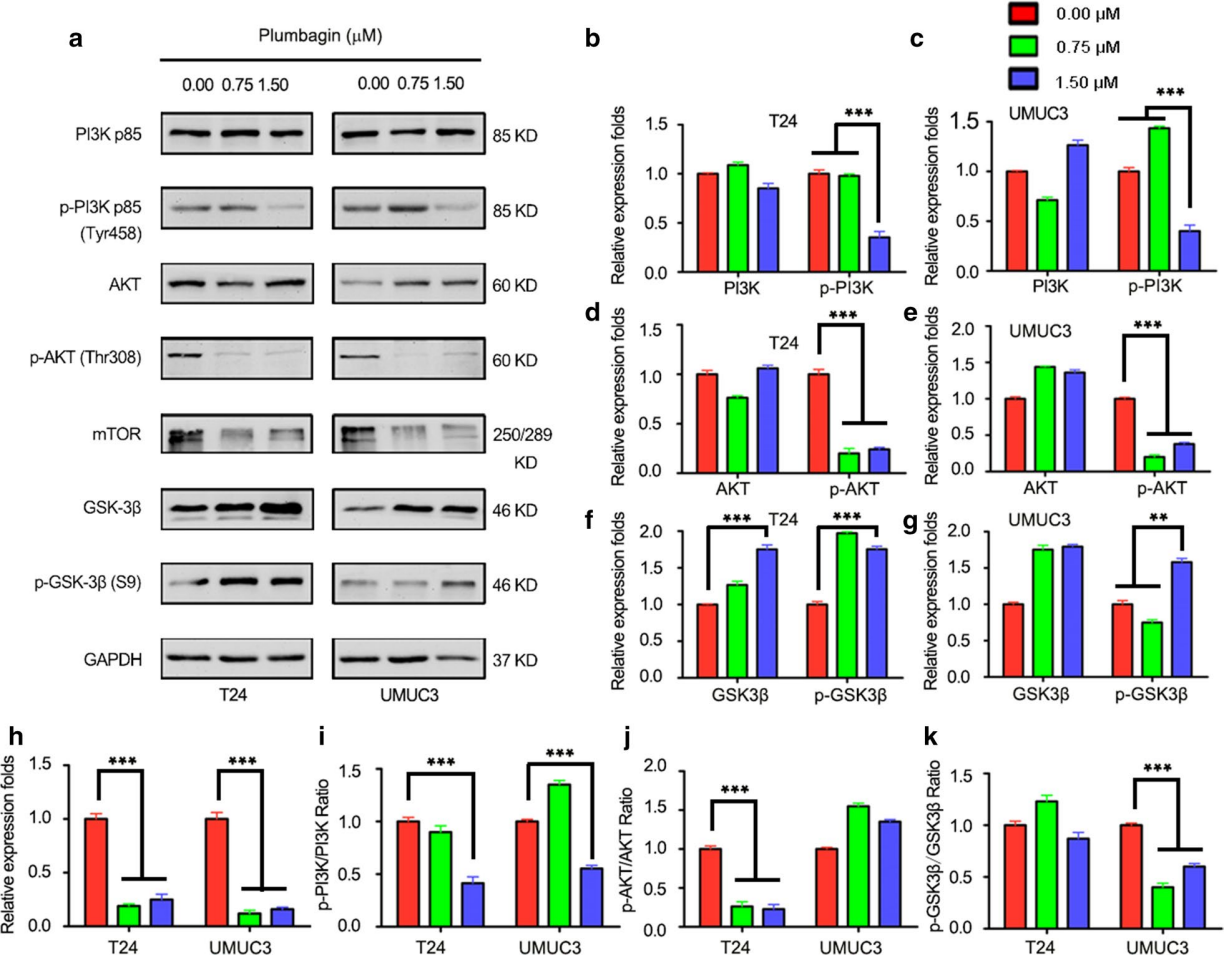
Effect of plumbagin on the PI3K/AKT/mTOR signal pathways in T24 and UMUC3 cells. **a** The expression levels of PI3K p85, p-PI3K p85, AKT, p-AKT, mTOR, GSK-3β, p-GSK-3β were determined by western blotting with plumbagin at 0, 0.75 and 1.5 μM. GAPDH was as the internal control; Bar graphs show the relative expression levels of PI3K and p-PI3K in T24 cells (**b**) and in UMUC3 cells (**c**); Bar graphs show the relative expression levels of AKT and p-AKT in T24 cells (**d**) in UMUC3 cells (**e**); Bar graphs show the relative expression levels of GSK-3β and p-GSK-3β in T24 cells (**f**) and in UMUC3 cells (**g**); Bar graphs show the relative expression levels of mTOR in T24 and in UMUC3 cells (**h**); **i**–**k** Bar graphs show the relative radio of p-PI3K/PI3K, p-AKT/AKT, p-GSK-3β/GSK-3β with plumbagin at 0, 0.75 and 1.5 μM. Data are the mean ± SD of three independent experiments. **P < 0.01, and ***P < 0.001

The potential mechanisms of cell cycle arrest and apoptosis were further investigated. First, we examined phosphorylation levels at Tyr458 of PI3K and phosphorylation at Thr308 of AKT, PI3K is activated and converted into PIP3 in cytomembrane, PI3K further binds to and phosphorylates AKT with a PH domain. When phosphorylated AKT is activated, it activates a variety of downstream signaling molecules to regulate cell proliferation, cell cycle, apoptosis, autophagy, and cell survival, Fig. 5 in this study shows results. Compared with untreated cells, phosphorylation of PI3K at Tyr458 was reduced by 65% in 1.5 μM PL while the total PI3K remain consistent for T24 cells with exposed to 0.75 μM and 1.5 μM PL, respectively (Fig. 5b); In UMUC3 cells, phosphorylation of PI3K at Tyr458 was reduced by 60% in 1.5 μM while total PI3K changed slightly from 0.75 to 1.5 μM PL (Fig. 5c). For p-AKT and AKT. Compared with the untreated group, total AKT changed slightly when cells were exposed to 0.75 μM and 1.5 μM PL, respectively. Phosphorylation of AKT at Thr308 decreased by 80.0% and 76.8%, respectively in T24 cells (Fig. 5d); Compared with the untreated group, Total AKT increased to some extent when cells were exposed to 0.75 μM and 1.5 μM PL, respectively. Phosphorylation of AKT at Thr308 decreased by 79.1%, and 62.3%, respectively in UMUC3 cells (Fig. 5e). Next, we calculated the ratio of phosphorylated protein to protein, the ratio of p-PI3K to PI3K was reduced by 11% and 59% in T24 cells treated with 0.75 and 1.5 μM PL for 24 h (Fig. 5i); In UMUC3, the ratio of p-PI3K to PI3K decreased by 55% in 1.5 μM (Fig. 5i). For the ratio of p-AKT to AKT, T24 cells treated with 0.75 and 1.5 μM PL decreased by 74% and 76% while UMUC3 cells had no apparent tendency (Fig. 5j). In addition, the total expression of the downstream protein mTOR decreased by 81% and 75% in T24 cells at concentrations of 0.75 and 1.5 μM PL; In UMUC3, 88% and 84% were reduced at concentrations of 0.75 and 1.5 μM PL (Fig. 5h). AKT activation in cancer could inhibit function of GSK-3β. As can be seen in Fig. 5f, the phosphorylation level of total protein and GSK-3β increased gradually. Specifically, In T24 cells, total GSK-3β protein increased by 26% and 75% and p-GSK-3β phosphorylation levels increased by 93% and 75% from 0.75 to 1.50 μM (Fig. 5f); In UMUC3 cells, total GSK-3β protein increased by 75% and 79% and p-GSK-3β phosphorylation levels increased by 58% in 1.50 μM (Fig. 5g), Fig. 5k shows that the ratio of p-GSK-3β to GSK-3β in T24 cells changed slightly, while in UMUC3 cells, the ratio of p-GSK-3β to GSK-3β increased by 60% and 40% in 0.75 and 1.5 μM, This indicated that PL could inhibit AKT activation to promote the phosphorylation of GSK-3β further play its role in cell proliferation and glycogen metabolism. In Conclusion, PL with different concentrations can play the role of anti-cancer cells by regulating the expression of downstream effectors in the PI3K/AKT/mTOR pathway.

### PL suppressed tumorigenesis in vivo

The next step is to explore the effect of PL in vivo. A BCa cell xenograft model was fabricated by subcutaneously injecting T24 cells into the treated mice, the PL treated group (PL) was injected intravenously with PL working solution every third day, the dose of PL for in vivo injection was set to be 10 mg/kg. The blank control group (B.C.) was injected with the same dose of ethanol solution. After injected for 10 times, the tumor samples were dissected for characterizations. Meanwhile, the organs of treated animals, such as heart, liver, spleen, lung, and kidney, were also harvested for staining.

**Fig. 6:**
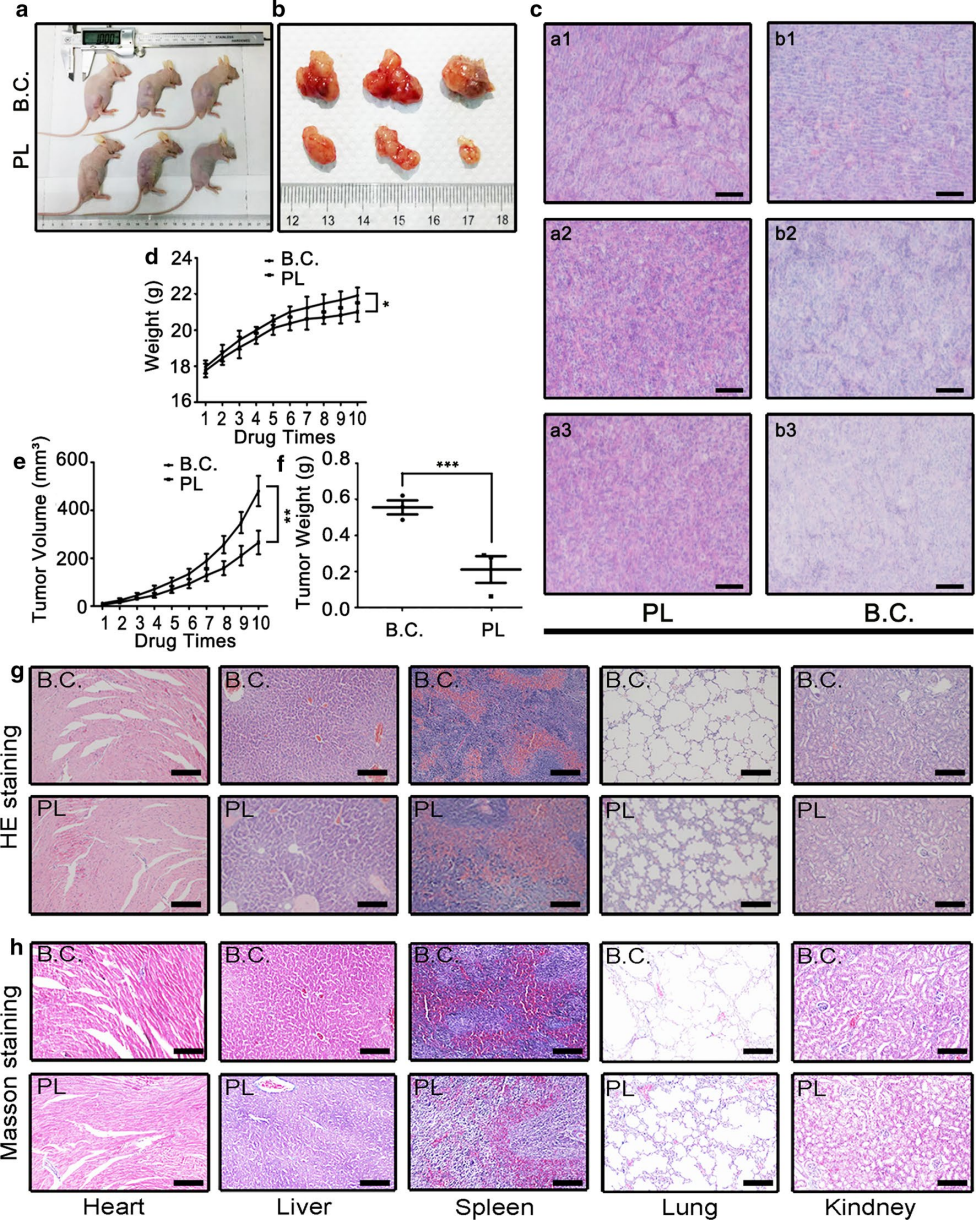
Plumbagin suppressed BCa tumorigenesis in vivo. **a**,** b** The photographs of tumor bearing mice and dissected tumor samples, respectively; **c** The HE staining images of tumor samples, a1-3 and b1-3 corresponding to the three individual animals in the PL group and B.C groups, respectively. Scale Bar: 100 μm; **d** the growth curves of animal weight during the whole period of drug injection; **e** the growth curves of tumor volume; **f** quantitative results of tumor weight; **g** representative HE staining images of the main organs (heart, liver, spleen, lung and kidney) dissected from the treated mice; **h** representative Masson staining images of the main organs. Scale Bar: 100 μm. *P < 0.05, **P < 0.01, and ***P < 0.001

The object images of treated mice are shown in Fig. 6a and the images of regenerated tumor tissues are shown in Fig. 6b. It could be observed that the tumor sizes in the PL group were much smaller than those in the B.C. group. As shown in Fig. 6d, the weight of mice decreased slightly after PL treatment. This phenomenon could be attributed to the delayed growth of tumor samples (Fig. 6e). Compared with the B.C. group, the average tumor weight in the PL group significantly decreased from 0.57 ± 0.04 g to 0.21 ± 0.06 g (Fig. 6f). The H&E staining of tumor tissues also confirmed a definitive in vivo anti-tumor activity (Fig. 6c). The images of H&E staining of animal organs are shown in Fig. 6g and the images of Masson staining are shown in Fig. 6h. It could be observed that the edema of liver cells slightly increased after PL treatment, and the Masson staining deepened. Additionally, the lung tissue integrity was also slightly destroyed. No other typical histopathological changes could be observed.

## Discussion

Muscle-invasive bladder cancer is one of the most common malignant tumors in men [27]. Transurethral resection combined with radiotherapy and/or chemotherapy is the gold standard of MIBC treatment [28]. However, the rate of postoperative recurrence is very high, and the survival of MIBC patients is not satisfactory. Zhang et al*.* fellow up 87 high grade T1 BCa patients who were treated with bladder preservation surgery in Zhongnan Hospital of Wuhan University, and found that the 2-year postoperative recurrence rate was as high as 63.21% [29]. Currently, infusion chemotherapy drugs such as gemcitabine and BCG has been applied to inhibit BCa recurrence and improve survival [30]. Meanwhile, bladder perfusion allows drugs to reach the tissues in situ for maximum efficacy regardless of drug uptake or metabolic pathways, the search for drug therapy of bladder cancer continues to be unabated, exploring new drugs with high efficiency is brought to the forefront.

**Fig. 7:**
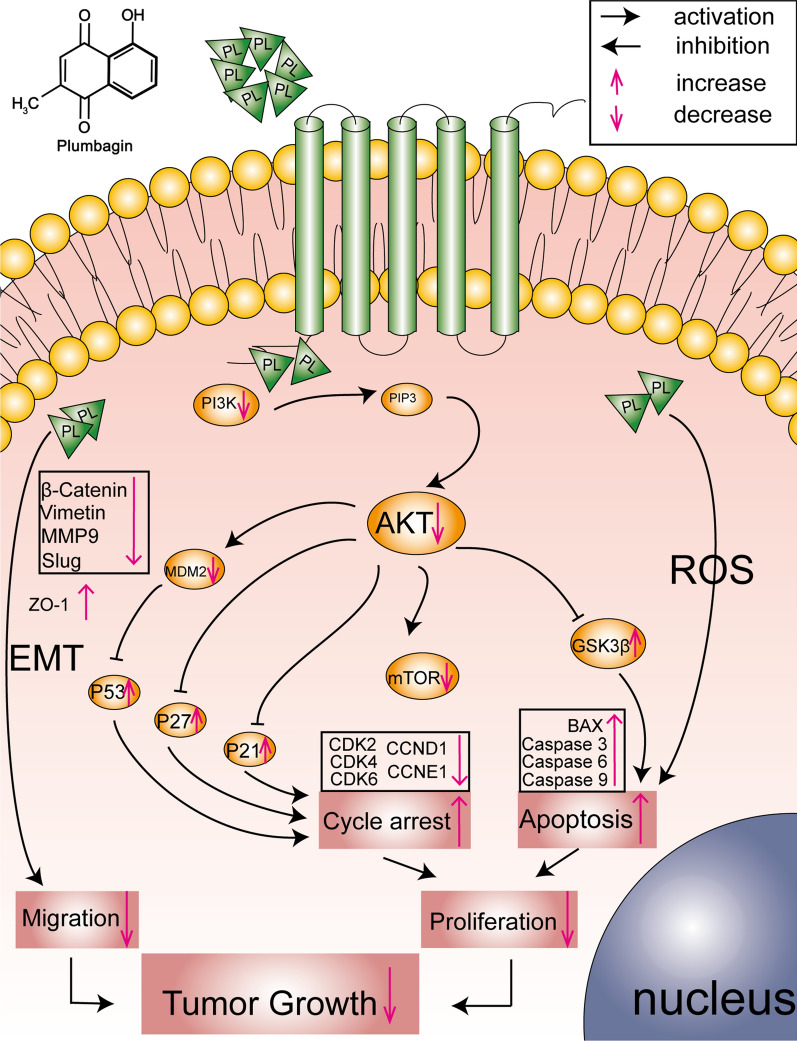
Potential pathway of anti-tumor activity of plumbagin in bladder cancer

Compared with traditional chemotherapy drugs, plant-derived drugs (PDDs) possessed the advantages of better efficacy and lower side effects [31]. In this work, PL was chosen as the candidate PDD for BCa chemotherapeutic treatment, and the application potential was investigated. PL, 5-hydroxy-2-methyl-1, 4-naphthoquinone, is a natural small molecule extracted from the root of *Plumbago* genus plants [32]. PL has been reported to be multifunctional and curable towards a variety of diseases, such as cancer and autoimmune disease [33]. Compared with the other plant-derived drugs, PL is lower in drug concentration, which is usually complicated with fewer side effects. In recent decades, the anti-tumor application of PL is of increasing interest. The anti-tumor mechanism of PL is complex and varies from cancer to cancer. According to previous literature, the main targets of PL include STAT3, NF-KB and p53. PL can inhibit the proliferation and survival for esophageal cancer cells by inhibiting STAT3-PLK1-AKT axis [34]; For breast cancer, PL was found to induce apoptotic cell death through the p53-dependent pathway in MCF-7 human cells [29]; Li Ting et al*.* reported that PL inhibited the liver cancer cells proliferation and induced apoptosis by down-regulating the expression of SIVA [35]; Wei et al*.* found that PL promoted the apoptosis of human hepatocellular carcinoma SMMC-7721 cells through caspase-3/vimentin signaling-mediated EMT [36]. These studies suggested that PL, a natural PDD extracted from Chinese herbal medicine, might be related to multiple targets and multiple pathways in anti-tumor effects. In our study, we firstly found that PL could inhibit the proliferation, migration and survival of BCa cells in vivo and in vitro through inhibiting PI3K/AKT/mTOR pathway and EMT in Fig. 7.

The malignant proliferation and terminal migration are the most critical biological processes for rapid progression and advanced metastasis in the tumor, they are the key evaluation directions for the effectiveness of chemotherapy, PL can inhibit the proliferation and migration of cancer cells with concentration and time dependence. MMP9, Slug, β-Catenin protein expression level significantly decreased and ZO-1 increased after drug stimulation at different concentrations. PL was proved to inhibit the migration of cancer cells through the EMT pathway. We further explored the mechanisms which PL inhibits tumor cell proliferation.

Various molecular events such as cell cycle, apoptosis, autophagy, cell metabolism and cellular immunity and microenvironment can inhibit cell proliferation in cancer cells. It is reported that PL can induce apoptosis and autophagy via sirtuin1- and PI3K/AKT/mTOR-mediated pathways in human prostate cancer cells [37]. Cell cycle and apoptosis are the most important ways to influence cell proliferation. We found that PL can down-regulate cyclin (CCND1 and CCNE1) and cyclin-dependent kinase (CDK2, CDK4 and CDK6) and down-regulate cyclin-dependent kinase inhibitor (p27 and p21) to effectively arrest the cell cycle in G1 phase rather than the G2 phase reported in most reports [38, 39], This is an interesting scientific question and worth exploring later. We also found that PL can induce apoptosis by up-regulating the expression level of pro-apoptotic factors such as BAX (mitochondrial pathway) and Caspase 9, Caspase 6 and Caspase 3, which are key factors in the process of promoting apoptosis. In addition, ROS including superoxide anion (O_2_−), hydroxyl free radical (OH) with hydrogen peroxide (H_2_O_2_) [40], we also found that PL caused oxidative damage and increased apoptosis by acting on cells with more ROS generation.

Protein kinase family plays an important biological function in the process of cell proliferation, growth, differentiation and apoptosis [41]. Increased AKT activity is closely associated with a variety of cancers [42]. PI3K/AKT/mTOR is one of the most key pathways in the protein kinase family [43]. Various growth factors and signal transduction complexes can convert PI3K into PIP3 by activating the receptor tyrosinase kinase, then PIP3 promotes the Ser308 of AKT. The activated AKT regulates the cell proliferation, survival, invasion and metastasis of tumor cells by phosphorylating multiple downstream factors such as mTOR, protein kinase and transcription factor. Our results showed that PL significantly reduced the expression of PI3K/AKT/mTOR at the protein level, therefore, we believe that PL may exert its cycle arrest and induce cell apoptosis through the PI3K/AKT/mTOR signaling pathway. AKT can promote the phosphorylation of MDM2 to inhibit the degradation of p53 [44], and AKT can directly inhibit the phosphorylation of p21 and p27 to achieve the purpose of cell cycle arrest [45, 46]. In addition, AKT inhibits the phosphorylation of GSK-3β [47], GSK-3β further induced apoptosis [48], several researchers have revealed that PL shows its cell cycle arrest and apoptosis by inhibiting PI3K/AKT/mTOR in other cell lines [49–51]. This is consistent with our results. Additionally, the organs, such as heart, liver, spleen, lung and kidney were dissected from the treated nude mouse, and then histological staining including H&E and Masson were carried out. The results showed a good anti-tumor effect in vivo.

### Current challenges and potential future perspectives

#### Limitations and disadvantages

As a novel drug in the progress of research and development, there are still several issues that must be considered for the following pre-clinical tests and applications. This work is carried out at the level of human BCa cells and nude mouse, and whether PL is suitable for humans remains to be proved by clinical trials.

### The emerging role of PL in the future

We screened out PL as a potential anti-tumor PDD for BCa chemotherapeutic treatment. There is a unique metabolic process for drugs entering the body. Thus, how to design a reasonable drug delivery system, and achieve maximum utilization need to be worked out [52, 53]. Nano-drug delivery system and liposome-drug delivery system, which can improve drug targeting, decrease the dosage of drugs and prolong the effective time of drug effect by slow-release effect, can be applied with PL. PL liposome produced by thin film hydration method with minor modifications has been proved to enhanced plasma half-life and therapeutic efficacy [54]. Developing a drug loading system for bladder cancer is our next assignment.

## Conclusions

In our study, we found PL inhibits the proliferation of T24 and UMUC3 cells in vivo and in vitro, which may play a role through several downstream effectors of PI3K/AKT/mTOR signaling pathway promoting the cell cycle arrest and apoptosis. Meanwhile, we consider that PL may inhibit the migration of bladder cancer cells via EMT suppression and induce ROS generation to make cell apoptosis. This work screened out PL with relatively good anti-tumor activity in human bladder cancer. It provides a reference for future clinical trials and new drug research and development, adds a choice for multi-drug combination to prevent drug resistance.

## Supplementary information

**Additional file 1: Figure S1.** PL inhibits proliferation and migration in T24 and UMUC3 cells. (a) Representative images of clonogenic BCa cells (T24 and UMUC3) after plumbagin treatment; (b) Quantitative analysis of clonogenic survival assay for T24 and UMUC3 cells; (c) The anti-migration activity of plumbagin for T24 cells was further evaluated using wound healing assay; (d) Quantitative analysis of wound healing assay for T24 cells. **Table S1.** Primer sequences used for qRT-PCR. **Table S2.** List of primary antibodies and secondary antibodies.

## Data Availability

All data generated or analyzed during this study are included in this published article and its Additional file 1.

## Abbreviations

PL: Plumbagin
DCFH-DA: The fluorescent probe 2′,7′-dichlorofluorescin diacetate
qRT-PCR: Quantitative real-time PCR
BCa: Bladder cancer
PDD: Plant-derived drug
BCG: Bacille Calmette-Guérin
EAU: European Association of Urology
LN: Lymph node
FDA: Food and Drug Administration
PI3K: Phosphatidylinositol 3-kinase
PIP3: Phosphatidyl inositol triphosphate
PDK1: Phosphoinositide dependent kinase-1
mTOR: Mammalian target of rapamycin
EMT: Epithelial–mesenchymal transition
FBS: Fetal bovine serum
DMSO: Dimethylsulfoxide
PBS: Phosphate buffer saline
DCFH-DA: 2,7-Dichlorofluorescin diacetate
MDM2: Murine Double Minute 2
GADD: Growth arrest and DNA damage
PVDF: Polyvinylidene fluoride
SDS-PAGE: Sodium dodecyl sulfate polyacrylamide gel electrophoresis
