# Supplementary material for: Suppressive effects of plumbagin on the growth of human bladder cancer cells via PI3K/AKT/mTOR signaling pathways and EMT
Source: Cancer Cell Int. 2020 Oct 27;20:520. doi: 10.1186/s12935-020-01607-y (PMC7590591; doi:10.1186/s12935-020-01607-y)
Supplement: Supplementary file 1 — Additional file 1: Figure S1. PL inhibits proliferation and migration in T24 and UMUC3 cells. (a) Representative images of clonogenic BCa cells (T24 and UMUC3) after plumbagin treatment; (b) Quantitative analysis of clonogenic survival assay for T24 and UMUC3 cells; (c) The anti-migration activity of plumbagin for T24 cells was further evaluated using wound healing assay; (d) Quantitative analysis of wound healing assay for T24 cells. Table S1. Primer sequences used for qRT-PCR. Table S2. List of primary antibodies and secondary antibodies. [file 12935_2020_1607_MOESM1_ESM.docx]

**Additional file**

**Additional Figures**


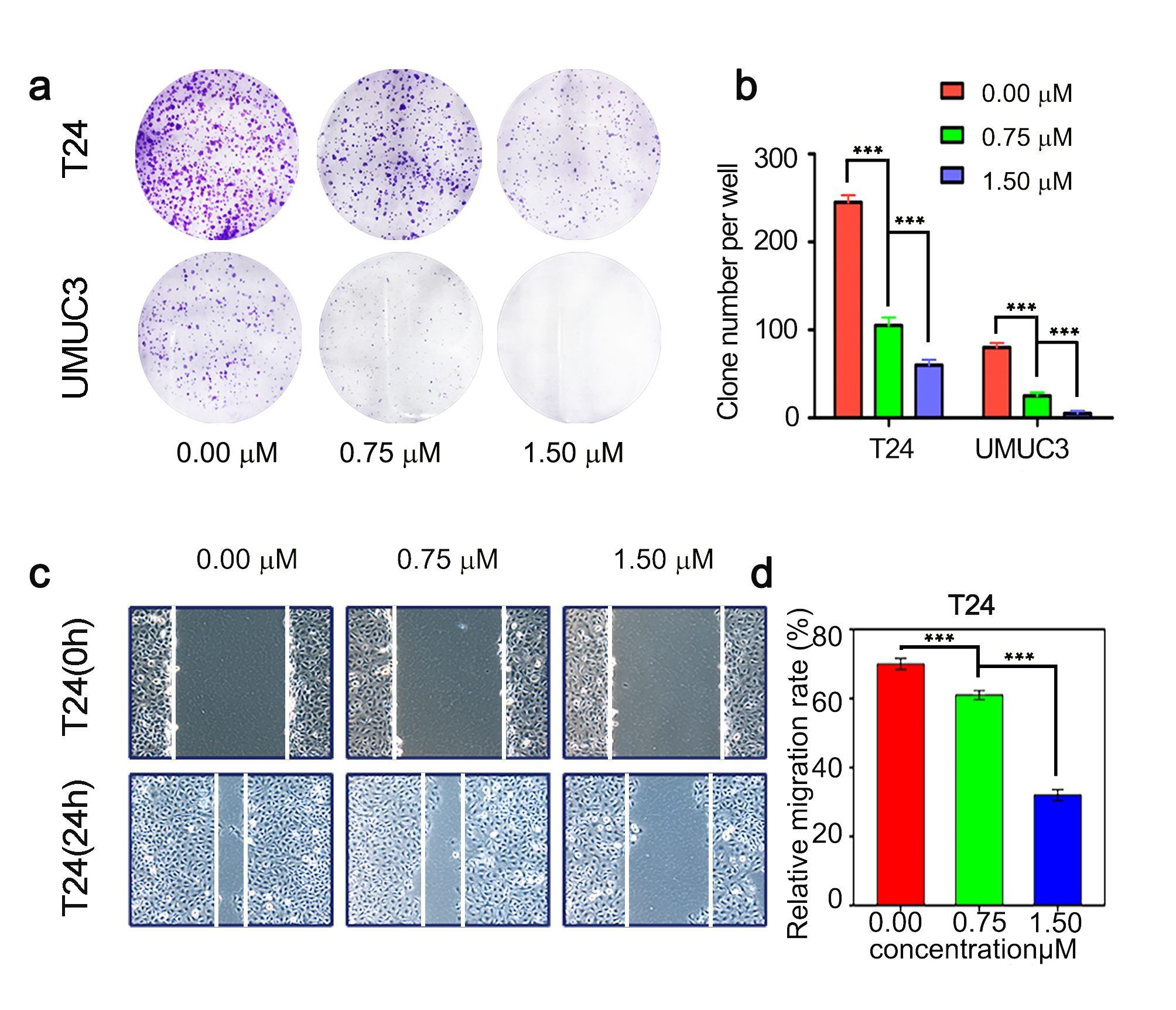


**Figure S1. PL inhibits proliferation and migration in T24 and UMUC3 cells. (a)** Representative images of clonogenic BCa cells (T24 and UMUC3) after plumbagin treatment; **(b)** Quantitative analysis of clonogenic survival assay for T24 and UMUC3 cells; **(c)** The anti-migration activity of plumbagin for T24 cells was further evaluated using wound healing assay; **(d)** Quantitative analysis of wound healing assay for T24 cells.

**Additional Tables**

**Table S1. Primer sequences used for qRT-PCR**

| Primer name | sequences 5’-3’ | Tm |
| --- | --- | --- |
| GAPDH-F | AATGGACAACTGGTCGTGGAC | 62.3 ºC |
| GAPDH-R | CCCTCCAGGGGATCTGTTTG | 61.6 ºC |
| MDM2-F | ATTCACTCAGCCAACCAGATCA | 61.4 ºC |
| MDM2-R | GCTTTGCCAACTTTCGTCTTTTT | 60.9 ºC |
| GADD-F | TACGAGTCGGCCAAGTTGATG | 62.1 ºC |
| GADD-R | GGATGAGCGTGAAGTGGATTT | 60.4 ºC |
| P53-F | AGCTTGATCGCCTCTATAAGGA | 60.2 ºC |
| P53-R | CCCTCAGCTCATTAACACGCT | 62.1 ºC |
| CDK2-F | CCCAAAGCAAATACGCGGAG | 61.7 ºC |
| CDK2-R | TCTGGCATTCCGTTCCGTTTC | 62.9 ºC |
| CDK4-F | AAACTTGGAAATCCCGAGATTGC | 61.4 ºC |
| CDK4-R | CGAAACCAGTTCGGTCTTTCAA | 61.0 ºC |
| P21-F | ATGCCTTCCGATCAGTACGAG | 61.4 ºC |
| P21-R | CGACCACGTAGATAGTGCTGT | 61.3 ºC |
| CDK6-F | TCTTCATTCACACCGAGTAGTGC | 62.2 ºC |
| CDK6-R | TGAGGTTAGAGCCATCTGGAAA | 60.5 ºC |

**Table S2. List of primary antibodies and secondary antibodies**

| name | species | dilution | resources |
| --- | --- | --- | --- |
| GAPDH | Mouse | 1:1000 | Abcam, USA, Cat. # ab181602 |
| CDK2 | Rabbit | 1:1000 | Cell Signaling Technology, USA, cat. no. 2546 |
| CDK4 | Rabbit | 1:1000 | Cell Signaling Technology, USA, cat. no. 12790S |
| CDK6 | Rabbit | 1:1000 | Cell Signaling Technology, USA, cat. no. 13331 |
| CCND1 | Rabbit | 1:1000 | Cell Signaling Technology, USA, cat. no. 2978 |
| CCNE1 | Rabbit | 1:1000 | Abcam, USA, Cat. # ab33911 |
| CCNB1 | Mouse | 1:1000 | Cell Signaling Technology, USA, cat. no. 4135S |
| P53 | Rabbit | 1:1000 | Proteintech, China, Cat. #10442-1-AP |
| P21 | Rabbit | 1:1000 | Abcam, USA, Cat. #ab109520 |
| P27 | Rabbit | 1:1000 | Abcam, USA, Cat. #ab32034 |
| β-Catenin | Rabbit | 1:1000 | Cell Signaling Technology, USA, cat. no. 8480T |
| Vimentin | Rabbit | 1:1000 | Cell Signaling Technology, USA, cat. no. 5741 |
| MMP9 | Rabbit | 1:1000 | Cell Signaling Technology, USA, cat. no. 13667 |
| Slug | Rabbit | 1:1000 | Cell Signaling Technology, USA, cat. no. 9585S |
| ZO-1 | Rabbit | 1:1000 | Cell Signaling Technology, USA, cat. no. 8193P |
| BAX | Rabbit | 1:1000 | Cell Signaling Technology, USA, cat. no. 5023S |
| Caspase 9 | Rabbit | 1:1000 | Cell Signaling Technology, USA, cat. no. 9508P |
| Caspase 6 | Rabbit | 1:1000 | Cell Signaling Technology, USA, cat. no. 9762 |
| Caspase 3 | Rabbit | 1:1000 | Proteintech, China, Cat. #19677-1-AP |
| AKT (pan) | Rabbit | 1:1000 | Cell Signaling Technology, USA, cat. no. 4691L |
| Phospho‐AKT(Thr308) | Rabbit | 1:1000 | Cell Signaling Technology, USA, cat. no. 9275L |
| PI3 Kinase P85 | Rabbit | 1:1000 | Cell Signaling Technology, USA, cat. no. 4257 |
| Phospho-PI3K P85(Tyr458)/P55(Tyr199) | Rabbit | 1:1000 | Cell Signaling Technology, USA, cat. no. 4228S |
| mTOR | Rabbit | 1:1000 | Abcam, UK, cat. no. Ab32028 |
| GSK3β | Rabbit | 1:1000 | Cell Signaling Technology, USA, cat. no. 12456S |
| Phospho‐GSK‐3β (Ser9), | Rabbit | 1:1000 | Cell Signaling Technology, USA, cat. no. 5558 |
| Anti-Mouse-IgG (H+L)-HRP | Goat | 1:10000 | Sungene Biotech, China, Cat. #LK2003 |
| Anti-Rabbit-IgG(H+L)-HRP | Goat | 1:10000 | Sungene Biotech, China, Cat. #LK2001 |
